# Knowledge, perception and experience of sexual entrapment among undergraduate students of the University of Ibadan, Nigeria

**DOI:** 10.12688/gatesopenres.12954.1

**Published:** 2019-05-15

**Authors:** Aisha I. Sule, Musibau A. Titiloye, Oyedunni S. Arulogun

**Affiliations:** 1 Department of Health Promotion and Education, University of Ibadan, Ibadan, Oyo, 200284, Nigeria

**Keywords:** Sexual entrapment, Harassment, Abuse, Assault, University, undergraduates, Perception, Experience, Nigeria.

## Abstract

**Background: **Deceit into sexual activities without the victim being aware of the intended action is common in tertiary institutions as the environment promote activities that make students vulnerable, especially females, young and new students. The resulting physical, psychological and social consequences, including harassment, sexual assault, non-consensual sex, injury, psychological trauma and suicide attempts, have affected many young people. This study was aimed at exploring the knowledge, perception, experience of sexual entrapment among undergraduate students of the University of Ibadan, Ibadan, Nigeria.

**Methods: **This multi-stage cross-sectional survey involved 422 participants using semi-structured questionnaire and an in-depth interview guide. Quantitative data were analyzed statistically, while Qualitative data was analyzed thematically.

**Results: **Mean age of respondents was 20.5±3.0 years, 52.6% were males, 58.8% and 81% had a good knowledge and perception of sexual entrapment, respectively. Prevalence of sexual entrapment was 18%, affecting more males (55.3%) and first year students (39.5%), 59.2% of the perpetrators are friends of the victim. Students were entrapped on campus through; emotional support (42.1%), money (34.2%), accommodation assistance (7.9%), material things/gifts (21.1%), assignment assistance (10.5%), help with registration (4.0%), religious activities (6.6%), debt repayment (11.8%) and counseling (9.2%). It resulted to; unwanted sex (34%), rape (4%), pregnancy (1.3%), STDs (4%), Injury (13%), psychological trauma (27.6%), loss of trust (52.6%) and attempted suicide (23.7%) among victims. Effective coping mechanisms were adopted by 55.3% of respondents. There was a significant association between knowledge and perception; knowledge and experience; perception and experience (p<0.05). Findings from the in-depth interview revealed care, tutoring, political positions, and pretense to need help as other tactics.

**Conclusions: **Sexual entrapment is common on campus, affecting males and females, knowledge and perception influence experience of sexual entrapment, requiring the need for a holistic approach to reduce its prevalence.

## Introduction

Entrapment entails inducing persons not disposed to carry out an activity to carry out such activity or an act of deception to commit an act or crime. For sexual entrapment, an individual is lured to sexual act unwillingly or through deceit using several means and strategies ^1^. Sexual entrapment is also a form of sexual abuse resulting in sexual harassment, involving an individual requesting for sex after rendering a gesture without the victim being aware of the intended action ^2^.

The vulnerability of young persons, a lack of psychological maturity and the grooming process employed by the perpetrators renders them powerless to recognize the exploitative nature of the relationship and inability to give informed consent ^2, 3^. Initially, perpetrators present themselves as empathetic and compassionate friends who offer help in difficult or straining situations ^4, 5^. A young person that desperately needs help can easily be seduced by the perpetrator’s fraudulent promises, help, safety and attention ^6^. Tactics resembling coercive and controlling techniques, such as; romancing and spending money; building dependence and/or trust by helping; isolation; abduction and/or drugging; isolation; coercion by financial or debt bondage; recruitment by gang; manipulation due to intellectual disability and normalizing sex, are also commonly used for sexual entrapment as observed in other types of exploitative relationship ^3^.

The university environment provides many opportunities for sexual activity, and first-year students, many of whom are classified as young people, are the most vulnerable to sexual entrapment due to their lack of experience to make good, risk-aware decisions about sexual liaisons as they are new on campus, there are many persons that may offer to help them through many activities and needs, such as the screening process, admission procedures, registration process, feeding, assignments and accommodation, among other things ^7^.

The impact and scope of sexual harassment that may be a result of sexual entrapment in colleges and universities surfaced in the early 1980s, leading to the creation of policies, procedures, extensive training programs and materials designed to identify and prevent sexual harassment ^8^. Despite these efforts to minimize or eradicate sexual harassment on campuses, the frequency of complaints is increasing ^8^. About two-third of university students have been sexually harassed and one-third of first year (41%) have been sexually entrapped and harassed by their peers ^9^. Age, year in school, gender, sexual orientation and familiarity has been identified as some of the risk factors associated with sexual entrapment on university campuses ^10– 13^.

Sexual entrapment, which may result in sexual harassment leading to unwanted sexual intercourse, threatens the victim’s sense of identity, thereby making it difficult for the individual to concentrate on studies, engage in promiscuous activities, depression, loss of trust, etc. ^14^. Victims of sexual entrapment that results in rape may experience emotional, social and sexual problem as a result of being sexually assaulted. Some suffer severe injuries, contract sexually transmitted diseases (STDs) or even get pregnant ^15^.

## Methods

### Study design and scope

A cross-sectional study design was used to determine the knowledge, perception and experience of Sexual entrapment among undergraduate students of University of Ibadan.

### Study setting

The study was carried out at University of Ibadan, the oldest university in Nigeria. The campus is located in the city of Ibadan (5miles i.e. 8 kilometers from the centre of the city), the capital of Oyo state, in south western Nigeria. The institution occupies over 1,032 hectares of land and was originally established on the 17th of November, 1948 as an external University College of the University of London. It was called University of Ibadan in 1962 and had over 12,000 undergraduate and post graduate students at that time. During the study, the institution had 13 faculties which include; Arts, Education, Law, Basic Medical science, Clinical sciences, Pharmacy, Public health, Dentistry, Veterinary Medicine, Technology, Agricultural sciences, Sciences and Social sciences. The University of Ibadan has 12 halls of residence, but there are 10 halls of residence for undergraduate students with 7 for males, namely Bello, Tedder, Mellanby, Independence, Nnamdi Azikiwe and Kuti, 3 for females, namely Queen Elizabeth II, Obafemi Awolowo and Queen Idia and one for both males and females, Alexander Brown Hall. The halls of residence have a caring capacity of over 8,000 students. The university has several social facilities that that can aid as a medium to sexually entrap on campus, including; the zoological garden, swimming pool, the university conference center, canteens and eateries, recreation center, cafeterias among others.

### Study population

The study population consisted of regular undergraduate students residing in the halls of residence within the campus. Regular students residing in halls of residence who accept to participate in the study by giving written informed consent were recruited for the study. Students who reside outside campus and those who did not consent to participating in the study were excluded from the study.

### Sampling procedure

A four-stage multi-stage sampling technique involving proportionate sampling at each stage was used to select 422 participants from all the halls of residence by randomly selecting one block from each hall and using proportionate sampling to get the specific number of participants to be selected from each hall and systematically selecting rooms from each block in August, 2017. Individual participants present in their room and who gave their informed consent at the time of recruitment were included in the study. The sample size was estimated using Leslie Kish’s formula n=Z ^2^pq/d ^2 ^with prevalence of 50% as there is no published prevalence of Sexual entrapment. Potential bias was controlled through effective training of research assistants and blinding them to the hypotheses of the research.

### Methods and instrument for data collection

A quantitative (questionnaire) and qualitative method (in-depth interview) were used for data collection. The questionnaire explored socio-demographic characteristics of respondents, knowledge, perception, experience, reaction and coping mechanism towards sexual entrapment. The in-depth interview further explored the perception and experience of sexual entrapment among those that had experienced it. The questionnaire and interview guide are available as *Extended data* ^16^.

### Procedure for data collection

Copies of the questionnaire were administered to selected study participants by the researcher and the four trained research assistants after obtaining informed consent through provision of adequate information about the study; on the purpose, the risk involved, the benefit and the requirement of the participant. Data was collected in the selected rooms of the study participants after the day’s lectures between 4.30 pm and 7:00 pm in August, 2017. The copies of the questionnaire were retrieved immediately after completion and checked to ensure they were correctly filled. The completed copies of the questionnaire were quickly reviewed to identify those that had experienced sexual entrapment for an in-depth interview. Verbal informed consent was sought from those identified to conduct in-depth interview immediately at their convenience to gain more insight of their experience. Verbal consent was obtained as written informed consent had been obtained previously. This was done in their room or a place that ensured convenience and privacy within the hall of residence. Each interview took a minimum of 10 minutes. The Audio recording and note taking was carried out during the interview sessions for those who consented to have a detailed interview.

### Validity and reliability of instrument

Validity of the instrument was ensured through consultation of relevant literature and subjection of the drafted questionnaire to critical review. The instrument was pre-tested among 10% (n=42) of the sample size at Obefemi Awolowo University, Ile-Ife undergraduate students due to their similar characteristics with the study population. This took place in July, 2017. Participants were selected randomly from 4 halls of residence and only those who gave written informed consent participated in the survey. Among the 42 participants that filled the questionnaire, three consented to the in-depth interview (2 females and 1 male). The data was inputted and analyzed using SPSS Version 22, reliability was obtained from Cronbach’s alpha. A reliability coefficient of 0.97 was obtained for this study, which indicated strong reliability of the instrument.

### Ethical considerations

Ethical approval was obtained from the University of Ibadan/University College Hospital Ethics Review Committee. An informed consent was sort from each research participant and confidentiality of study participants were adequately ensured.

### Data management, analysis and presentation

Data collected through questionnaire was serially numbered, coded and cleaned for errors by one person. The coding tree involved allotting numbers to responses to a particular question e.g. Yes= 1. No = 2, No response = 3 etc. Descriptive and Chi-square statistics were done using Statistical Package for Social Sciences (SPSS) version 22 software at p<0.05. The in-depth interviews sessions were transcribed and analyzed manually using thematic approach. The themes were identified based on general response to each question.

## Results

### Socio-demographic characteristics

The ages of the respondent ranged from 16 to 43 years with a mean of 20.5±3.0 years. Majority of the respondents (59.7%) were aged between 16 and 20 years. The majority (52.6%) were male and those in year one had higher number of participants (26.3%). Most of the respondents were Christians (85.3%). Yorubas as a tribe constituted the majority of the respondents (78.2%) and a majority (87.9%) were from monogamous families ( Table 1). Demographic information, alongside answers to the questionnaire and interview as well as a coding guide, are available as *Underlying data* ^16, 17^.

**Table 1.** Socio-demographic characteristics of respondents.

Socio-demographics	Frequency	Percentage
**Age in Years**		
≤20	237	59.7
21–30	156	39.3
31–40	3	0.8
≥41	1	0.2
**Sex**		
Male	222	52.6
Female	200	47.4
**Level of study**		
100	111	26.3
200	97	23.0
300	66	15.6
400	94	22.3
500	41	9.7
600	13	3.1
**Religion**		
Islam	55	13.0
Christianity	360	85.3
Traditional	5	1.2
Atheist	2	0.5
**Ethnicity**		
Yoruba	330	78.2
Igbo	63	14.9
Hausa	1	0.2
Others *	28	6.7
**Family of Origin**		
Monogamy	371	87.9
Polygamy	38	9.0
Single parent	13	3.1

*Other tribes: Edo (11), Efik (1), Ijaw (1), Gbagyi (1), Okrika (1), Urhobo (2), Tiv (1), Idoma (2), Ebira (4), Igala (2), Edoborn (1) and Etulo (1).

### Knowledge of respondents on sexual entrapment

Generally, 58.8%, 39.1% and 2.1% had a good, fair and poor knowledge of sexual entrapment, respectively. Knowledge on different aspects of sexual entrapment indicated that 72.5% had good knowledge of the different modes or scenarios of sexual entrapment and 71.6% had good knowledge on the different tactics used for sexual entrapment. In relation to the causes, victims, perpetrator, experience and consequences of sexual entrapment, 54.0% had good knowledge ( Table 2).

**Table 2.** Knowledge on different aspects of Sexual entrapment (N=422).

Knowledge	Level of knowledge		
	Good, n (%)	Fair, n (%)	Poor, n (%)
General knowledge of sexual entrapment	248 (58.8)	165 (39.1)	9 (2.1)
Definition, Modes/methods of sexual entrapment	306 (72.5)	89 (21.1)	27 (6.4)
Tactics used for entrapping students on campus	302 (71.6)	109 (25.8)	11 (2.6)
Causes, Victims, Perpetrators, Experience and Consequences of Sexual entrapment	228 (54.0)	185 (43.8)	9 (2.2)

### Perception of respondents on sexual entrapment

Perception of sexual entrapment revealed that only 40.8% of participants perceived sexual entrapment as being serious in the society and campus community; 44.1% perceived some categories of persons as being susceptible to sexual entrapment and some factors to make individuals susceptible to sexual entrapment; 83.4% perceived sexual entrapment as being a threat to students on campus; 89.3% perceived sexual entrapment as being beneficial in satisfying sexual desires and 83.2% agreed to the suggested perceived ways of preventing sexual entrapment as presented in Table 3.

**Table 3.** Perception towards various aspects of sexual entrapment (N= 422).

Perception	Perception level	
	Good, n (%)	Poor, n (%)
Perceived seriousness of sexual entrapment	172 (40.8)	250 (59.2)
Perceived susceptibility of certain categories of people to sexual entrapment and different factors that might make an individual susceptible to sexual entrapment	186 (44.1)	236 (55.9)
Perception of Sexual entrapment as a threat	352(83.4)	70(16.6)
Perception of Sexual entrapment as being beneficial	377(89.3)	45(10.7)
Perceived ways of preventing sexual entrapment	351(83.2)	71(16.8)
General perception on sexual entrapment	342(81.0)	80(19.0)

*Multiple responses included

### Experience of sexual entrapment

Among the 422 respondents, 76 (18%) have experienced one form of sexual entrapment or another. Of the total respondents, 10.9% revealed that they were asked for sex in return for the help they had received. In determining the various tactics used for sexual entrapment, it was observed that more of the participants (42.1%) were lured through emotional support. In relation to determining the position/relationship of the perpetrators to the victims of sexual entrapment, the majority (59.2%) were entrapped by their friends, 22.4% by their classmates, 17.1% by a senior student, 5.3% by a religious fellowship member, 4% by a lecturer, 4% by non-academic staff and 9.2% by people outside the school environment including relatives and neighbors. 40.8% of the respondents’ experienced sexual entrapment before coming to the university followed by 39.5% while in 100 level ( Table 4).

**Table 4.** Experience of sexual entrapment (N=422).

Experience of sexual entrapment	Frequency	Percentage
Ever experienced sexual entrapment	76	18.0
**Forms of sexual entrapment**		
I have ever been in a situation where someone asked me for sex in return for the help I had received	46	10.9
I was in a position where i couldn’t say no to sexual advance	37	8.8
Someone deceived me into having sexual intercourse by pretending to be nice	27	6.4
My partner abandoned me immediately we had sexual intercourse because that was his goal unknown to me	18	4.3
Someone wanted to or had sex with me because he/she was in a position of helping me out of trouble	11	2.6
I was asked to offer sex because I could not pay up a debt	10	2.4
Someone deceived me into a sexual relationship after helping my spiritual life	7	1.7
**Tactics used for sexual entrapment**		
I was entrapped through help with emotional support	32	42.1
Money was used to entrap me	26	34.2
Material things/gift was used to entrap me	16	21.1
I was entrapped because i was indebt	9	11.8
I was entrapped through help with assignment	8	10.5
I was entrapped through help with Counseling	7	9.2
Accommodation was used to entrap me	6	7.9
I was entrapped through help with spiritual intercession	5	6.6
I was entrapped through help with registration	3	4.0
**Age of experience of sexual entrapment**		
18 years and below	52	68.4
Above 18 years	24	31.6

### Outcome of sexual entrapment

Among the 76 respondents that experienced sexual entrapment, it resulted in sexual intercourse among 34%, 4% were raped, 13.2% acquired injury while trying to escape from being raped, 1.3% became pregnant, 4% discovered they had contracted an STD. The majority (66%) managed to escape from having sex with the perpetrator. A majority (52.6%) revealed that they lost trust in people, 44.7% were very angry, 38.2% felt embarrassed, 32.8% were afraid, 27.6% suffered psychological trauma and 19.7% lost their self-esteem ( Table 5).

**Table 5.** Outcome of sexual entrapment.

Outcome of sexual entrapment	Frequency	Percentage
**Physical outcome**		
I managed to escape from having sexual intercourse with the person that sexually entrapped me.	50	66.0
I had unwanted sex with the person that sexually entrapped me	26	34.0
I acquired injury from trying to escape from being raped	10	13.2
I was raped by the person that sexually entrapped me	3	4.0
I contracted sexually transmitted infection(s)	3	4.0
I became pregnant	1	1.3
**Psychological outcome**		
I lost trust in people	40	52.6
I was angry	34	44.7
I felt embarrassed	29	38.2
I was always afraid	25	32.8
I suffered from psychological trauma	21	27.6
I lost my self-esteem	15	19.7

### Coping mechanism adopted after being sexually entrapped

Among the 76 participants, 46.1% avoided relationship with people, 26.3% later had a good relationship with the perpetrator, 44.7% talked to someone about it, 55.3% tried to forget about the incidence, 18.4% sought medical attention, 39.5% avoided activities for a while, 23.7% attempted suicide, 17.1% reported to the school authority, 36.8% resulted to risky sexual practices, 27.6% resulted to entrapping others and 22.4% eventually harmed the perpetrator as presented on Table 6. The majority (55.3%) possessed an effective coping mechanism after been sexually entrapped.

**Table 6.** Coping mechanism adopted after being sexually entrapped.

Coping with sexual entrapment	Frequency	Percentage
I tried to forget the incidence *	42	55.3
I avoided relationship with people	35	46.1
I talked to someone about it *	34	44.7
I avoided activities for a while	30	39.5
I resulted to risky sexual practices	28	36.8
I resulted to entrapping others	21	27.6
I Had a good relationship with the person that sexually entrapped me	20	26.3
I attempted suicide	18	23.7
I eventually harmed the perpetrator	17	22.4
I went to a health centre for medical checkup *	14	18.4
I reported to the school authority *	13	17.1

*Positive responses

## Discussion

The result indicating that a good number of the respondents had a good knowledge of sexual entrapment (58.5%), a figure comparable to that observed in a study by Menon *et al*. ^15^. This could be attributed to the fact that sexual entrapment occurs on campus and most people would have come across or heard about someone that had experienced it, even though most of them were not familiar with the term. The level of knowledge also influenced their perception (p<0.05) as majority (81%) of respondents had a good general perception towards sexual entrapment, perceiving it as being serious, seeing it as a threat within the campus community and considering everyone on campus to be at risk. This is because most of them disagreed that not only females can be entrapped within the campus. This is also in line with the study by Menon, *et al.* ^15^.

This level of perception could be attributed to the fact that people see sexual entrapment as rampant within the campus, as there are several means, strategies and situation that can be used for sexually entrapping students ranging from distribution of phone numbers of females among some male students, using food joints and canteens and the freedom of the opposite sexes to visit each other’s rooms within the halls, among others. This finding, however, contradicts the statement that Nigeria as a society does not perceive the concept of harassment as evil or as a violation of human rights, as it is assumed that individuals have the right to express and satisfy themselves, which implies that sexual entrapment is also considered a norm and not a big deal ^8^.

According to Carina ^8^, most Nigerian communities believe in male supremacy; thus, sexual entrapment may be perceived as an acceptable exercise of a male’s prerogative over a female’s sexuality. This is, however, not in line with the present study, as a majority (81.5%) disagreed with manliness being a privilege or advantage for sexually entrapping females. This finding can be explained by the fact that the target population has acquired some level of education that has influenced their perception, maybe as a result of exposure to different media and circumstances where different forms of sexuality have been explained. They may also have witnessed or experienced sexual entrapment. Their level of education may have also influenced their perception to the fact that both males and females should have equal rights.

Even with the high levels of good perception observed in this study, some students considered sexual entrapment within the campus community as a norm and an event that should be expected within the campus. This finding was supported by a study that revealed that students perceive entrapping other students sexually to be a way of having fun, and just one of school’s escapades, while some perceive that the victims also wanted sexual attention and are responsible for being entrapped ^18^. This may be because the campus is seen as an environment that enables perpetration of such activities as a result of an absence of laws against it on campus and a lack of punitive measures within the campus. According to Okoro and Osawemen ^19^, many of them believed that sexual harassment generally was a norm in society, as they see their society as being unsafe, hostile and intimidating, and may alter their own behaviour in an attempt to decrease their sense of vulnerability and were of the view that the prevalence of sexual harassment may have resulted from sexual entrapment in the societies.

The prevalence of sexual entrapment amongst 18% of the participants (n=76), affecting 42 males and 34 females indicates that sexual entrapment is common among undergraduates of the University of Ibadan, although this result cannot be directly compared to any study conducted on sexual entrapment as this area has not been researched on independently, but have been incorporated as a means of sexual harassment (i.e. sexual entrapment has not been researched on as a single entity but has been indirectly identified as a means of sexual harassment in studies). In this regard, the prevalence is lower than that of a study conducted among undergraduate students in Udupi district, which revealed that enticing persons through gifts and rendering of help and services (which is a form of sexual entrapment constituted 45.5% of the various means to sexual harassment among the students ^20^. Sexual entrapment was observed to be higher in males than females, although this was not statistically significant (p>0.05). This finding contradicts most of the studies on sexuality generally, as it is expected that females are usually more affected than males as they are seen as vulnerable and the fact that culture has placed females on the receiving end of sexual advances, as it is believed that males are meant to approach females ^9^. However, this finding is similar to a study conducted among university students in Kenya, which revealed that more males were sexually harassed than females ^20^. The possible explanation for this finding could be attributed to the fact that times are changing and both males and females have sexual needs to meet, although the female perpetrators often use non-verbal cues like seduction after rendering help.

Asking for sex in return for help rendered especially emotional support was the mostly used tactics for sexually entrapping others on campus. This is supported by Reid *et al*. ^3^ that propounded coercive and controlling techniques used for sexual entrapment where independence and trust is built first by the perpetrator. This could be explained by the fact that a majority of the victims are young people who are often faced with one emotional/psychological problem after the other, including pressures in their relationships and academic and family life, and may need someone to talk to or confide in.

This study observed that victims were mainly sexually entrapped by their friends, classmates, senior students, fellowship member, lecturers and non-academic staff, people outside the university environment, such as relatives, neighbors, etc. This finding conforms to that of a study that revealed perpetrators are mostly male friends, partners, teachers and peers ^21^. The higher prevalence of sexual entrapment by friends can still be attributed to the fact that perpetrators try to gain the trust and love of their victims and become friends before opening up on their intentions ^3^. It was observed that the prevalence decreased with seniority of class, as a majority experienced it during their first year. Among tertiary institution students, attention has been paid to the “red zone”, (i.e. when students are new on campus) during which students are at perceived risk of experiencing sexual assault or entrapment. Evidence has been established that the start of school for first year students is the most risky time for students to be prone to sexual assault and entrapment, as they are new on campus and may need help to adapt to the school environment, and most of them are young and naïve ^22^. This finding is also in line with a study by Cranny, 2014 ^12^.

The study revealed that most of the victims of sexual entrapment were entrapped by the opposite sex, while few males were entrapped by the same sex and one person was entrapped by both the same and opposite sex. Our finding, that the same-sex form of sexual entrapment is more prevalent among males, is comparable to a study where one-quarter of males admitted harassing male students, compared to one-tenth of female students’ harassed female students in a nationally representative survey of undergraduate college students ^18^; i.e. males sexually entrap themselves more than females insinuating intended action of homosexuality on campus.

The findings of this study revealed that unwanted sexual intercourse with the perpetrator was the most frequently experienced negative outcome of being sexually entrapped, followed by acquiring injury while trying to escape from having sex with the perpetrator, rape, pregnancy after having sexual intercourse with the perpetrator and contracting STDs. This findings conforms to the report by Maharaj ^23^ highlighting the short/intermediate-term consequences of sexual entrapment in the form of unwanted touch, unwanted kiss, unwanted sexual intercourse, rape, etc. ^24, 25^. It resulted in sexual intercourse more in males than females because some of the males may be overtaken by emotion and seize the opportunity to explore and have sexual intercourse, since they may feel they have nothing to lose. The females on the other side are more at risk for the resulting consequences, and hence will try to fight to avoid any form of sexual intercourse, thereby resulting to high prevalence of escape from having sex with the perpetrator in females than males.

In exploring the various ways in which victims of sexual entrapment tried to cope and deal with the experience and the various actions taken aftermath, 55.3% of victims adopted an effective coping mechanism, such as talking to someone about it, going to the health center for a medical checkup and reporting the incident to a school authority, while the rest adopted ineffective coping mechanisms, such as avoiding relationship with people, avoiding activities, attempting suicide, resulting to sexual risky practices, resulting to sexually entrapping others and harming the perpetrator. This finding relates to the modes of reactions established by Michele *et al*. ^26^, which include advocacy/help seeking, social coping (i.e. seeking support from friends), avoidance/denial (i.e. trying to ignore what happened) and confrontation/negotiation (i.e. trying to deal directly with the perpetrator).

## Conclusion and recommendations

Sexual entrapment is common among undergraduate students of the University of Ibadan surveyed, and affects both male and female. Younger and first year students are most affected. Young people should mindful of the kind of friends they keep and parents should establish a good relationship and always educate their children starting from a younger age to be able to detect exploitative relationships. 

## Data availability

### Underlying data

Harvard Dataverse: Knowledge, perception and experience of Sexual entrapment among Undergraduate Students of the University of Ibadan, Nigeria. https://doi.org/10.7910/DVN/7XXADC ^16^.

Sule *et al*. data file.tab contains answers to each question from each participant.

Harvard Dataverse: Knowledge, perception and experience of sexual entrapment among undergraduate students of the University of Ibadan. https://doi.org/10.7910/DVN/L2S1R2 ^17^.

Coding guide for Knowledge, perception and experience of sexual entrapment among undergraduate students of the University of Ibadan.docx contains a coding guide for the above dataset.

### Extended data

Harvard Dataverse: Knowledge, perception and experience of Sexual entrapment among Undergraduate Students of the University of Ibadan, Nigeria. https://doi.org/10.7910/DVN/7XXADC ^16^.

Questionnaire and IDI guide.docx contains the questionnaire and interview guide used in this study.

Data are available under the terms of the Creative Commons Zero "No rights reserved" data waiver (CC0 1.0 Public domain dedication).
