# Supplementary material for: Knowledge, perception and experience of sexual entrapment among undergraduate students of the University of Ibadan, Nigeria
Source: Gates Open Res. 2019 May 15;3:1466. [Version 1] doi: 10.12688/gatesopenres.12954.1 (PMC8901584; doi:10.12688/gatesopenres.12954.1)
Supplement: Peer review history

# Peer review: Reviewer response for version 1

**DOI:** 10.21956/gatesopenres.14059.r31790

**Published:** 2022-03-15

**Authors:** Laila Rahman

**Affiliations:** 1 University of Western Ontario, London, ON, Canada

**General comments:**

 This article took me to an interesting path to understand the phenomenon of sexual entrapment among the undergraduate students in a Nigerian university. Based on a cross-sectional survey conducted among the undergraduates, the authors presented the prevalence of sexual entrapment among the students, the consequences they faced, and the coping mechanisms they employed. This research has generated questions for future research and contributed to the scholarship on sexual entrapment.

 However, in navigating this article, I stumbled upon several times with a few concerns and questions that need clarification. Addressing these questions might improve the transparency and rigor of this interesting work.   

**Specific comments:**

***Piquing readers’ interest:***

 The Introduction section of any research article has to do the heavy lifting of piquing readers’ interest by responding to their implicit question: why should the readers care for this particular research?

 I am afraid that this article’s introduction and abstract’s background sections fell short in answering this question. The abstract had a good start as it informed readers that students in tertiary institutes commonly experience sexual entrapment which has severe consequences for them. Then, without highlighting the gap in the literature or value for their research, the authors presented the research aim that would apparently produce information already known to them. It would have been helpful if they had articulated the connection that bridges the research problem with the research aim. For example, it would have been helpful to read statements along the following lines:

 "...Sexual entrapment, growing out of the sexual trafficking literature, has not been explicitly studied among the university students. Examining sexual entrapment might help understand this specific phenomenon and facilitate students and universities to reduce student sexual exploitation in the future. Therefore, this study aims to..."     

 Curiously, the authors started the introduction section with a definition of entrapment and ended with the consequences of sexual entrapment for students. Before delving into the methods section, readers need to be persuaded about the aims and value of this research. For this, they need to know the research problem (i.e., sexual entrapment) for the relevant population group (i.e., undergraduates) in a particular research setting (i.e., a Nigerian university) in order to be convinced that this problem can be (at least partially) remedied by taking up this research project.

 The authors have touched on some of these issues but left out others to readers’ imagination. They, for example, did not point out what was not known about this problem that needed to be known (by way of exploring the knowledge, perception, and experiences of sexual entrapment among the undergraduates). And how taking up this research could address the problem of sexual entrapment faced by students.

 Starting off the introduction section with texts similar to the abstract’s background section and ending with research aims/questions and the significance of this research might be helpful. Clarifying the value in invoking the concept of sexual entrapment rather than sexual harassment might also help readers to fully appreciate this innovative research.

***Conveying the meandering methods***

 Clearly conveying the methods, especially when it is meandering and complex, is always a challenge. However, as readers need to understand the methods to value the findings of any research initiative, clearly describing the methods is important.

 I am afraid that the sampling procedure, especially the four-stage sampling technique, was not at all clear. The authors might present a figure on sample selection that will start with the sample universe and end with the final selection of the sample. Specifying the numbers of non-responses and missing cases is also important to fully appreciate this study.

 Before presenting the validity and reliability of the study measure (by the way, what measure? Sexual entrapment measure?), the authors also need to provide a description of outcome and explanatory variables in a sub-section on Variables. Merely providing the questionnaire and code as supplementary materials does not replace the need for this information.

 While some information was left out, providing others such as the procedures of qualitative in-depth interviews without presenting their findings was misleading. I expected to read insights gained from these interviews, but they were not included in this article, right? Therefore, consider removing information about in-depth interviews from the abstract, and how they were analyzed from the methods section.  

***Ethics in researching violence and traumatic experiences***

 As recounting traumatic experiences may cause further discomfort and trauma, the readers need to know if the participants were provided with information on support services if they needed help to deal with such discomfort and trauma.    

***Presenting atypical findings***

 This study findings indicated that more men compared to their women counterparts had experienced sexual entrapment, although this difference, according to the authors, were not statistically significant. Given that sexual entrapment occurs between the opposite and within the same sex individuals, it would be better if the authors could provide further information, especially the prevalence rates across at least four categories of sexual entrapment: (a) men to women, (b) men to men, (c) women to men, and (d) women to women. 

***Doing research towards changing social structures and processes***

 The Discussion section needs to discuss the implications of this research for future research, policy, and intervention programs. As transformative research projects aim to change social structures and processes, the authors need to discuss what structures and processes in the universities and societies are conducive to sexual entrapment and how they might be disrupted to stop sexual exploitation against students in the future. 

***Reporting statistics with uncertainty***

 To acknowledge the uncertainty in statistical methods, the authors might present the 95% confidence intervals of different point estimates; and they should report the missing cases of age category in Table 1. The authors should avoid presenting the significance level at p<0.05 without an accompanying statistical estimate. All tables should have a note on the name, date, and sample size of the survey. All multiple responses should be identified in tables (e.g., forms of sexual entrapment in Table 4, physical and psychological outcomes in Table 5). As the difference between forms and tactics of sexual entrapment in Table 4 felt conflated, the authors might clarify their differences or present tactics only in texts or in a separate figure. In Table 6, instead of using asterisks (*), positive and negative coping mechanisms should be presented under separate categories similar to Table 5’s physical and psychological outcomes.   

***Playing the language game***

 Playing the language game well, among other things, is required to increase the uptake of any research. The authors need to closely read and re-read their article to edit the text. A few examples of such edits (purely for demonstration) are given below. The authors are advised to take help from a professional copy-editor to edit the full article, if possible.

*Example 1.*

 Original text: "Deceit into sexual activities without the victim being aware of the intended action is common in tertiary institutions..."

 Edited text: "Getting deceived into sexual activities is common in tertiary institutions."

 Explanation: The phrase, without the victim being aware of the intended action, is redundant as this idea is implied in the word ‘deceive.’

*Example 2.*

 Original text: "This multi-stage cross-sectional survey involved 422 participants using semi-structured questionnaire and an in-depth interview guide."

 Edited text: "This multi-stage cross-sectional survey involved 422 participants using semi-structured questionnaire and 76 participants using an in-depth interview guide."

 Explanation: Follow the parallelism in sentence construction.

*Example 3.*

 Original text: "Students were entrapped on campus through; emotional support,..."

 Edited text: "Students were entrapped on campus through emotional support,..."

 Explanation: A semi-colon is not appropriate to present a list of items.

*Example 4.*

 Original text: "There was a significant association between knowledge and perception; knowledge and experience; perception and experience (p<0.05)."

 Edited text:" Sexual entrapment knowledge was associated with sexual entrapment perception (chi-squared (degrees of freedom?, N = sample size?) = ?, p=?) and experience (chi-squared (degrees of freedom?, N = sample size?) = ?, p=?). Perception was also associated with experience (not shown)."

 Explanation: Using semi-colons instead of commas to separate a simple list of items is not appropriate. Statistical results need to be presented with evidence. P-values should not be presented without the associated estimates such as correlation, chi-squared or regression coefficient values.  

*Example 5.*

 Original text: "The university environment provides many opportunities for sexual activity, and first-year students, many of whom are classified as young people, are the most vulnerable to sexual entrapment due to their lack of experience to make good, risk-aware decisions about sexual liaisons as they are new on campus, there are many persons that may offer to help them through many activities and needs, such as the screening process, admission procedures, registration process, feeding, assignments and accommodation, among other things."

 Edited text: "The university environment provides many opportunities for sexual activity, and first-year students, many of whom are young people, are most vulnerable to sexual entrapment (Ref?). They fall into these traps, as they lack experience to make risk-averse decisions about sexual liaisons with people who offer them help. For being new on campus, they need support in going through the screening, admission, and registration processes, planning for eating and accommodation, and completing assignments among other things (Ref?). When people offer them help with an ulterior motive, they might fall into their traps of sexual exploitation."

 Explanation: The original text is one big, run-on sentence, expressing at least five ideas without appropriate use of connecting words. The authors might have wanted to avoid repeating the same reference. However, an ambiguous sentence that slows down readers is more problematic than a repeated reference.

*Example 6.*

 Original text: "....and one-third of first year (41%) have been sexually entrapped and harassed by their peers."

 Problems: One-third does not represent 41%, two-fifths does. And who is the subject in this clause? One-third of first year students? Please clarify.

***Weaving a credible web of references***

 In-text citations and references help authors to tell a story that is credible. This article has met with a few in-text citation and reference related problems. These include: (a) not providing a reference when it is warranted, (b) citing a reference for a claim that was not supported by the cited reference, (c) providing somewhat vague references in the reference list. Examples of these concerns are provided below.   

 (a) What is the reference for the first sentence of this article that depicted ‘entrapment’? As this information is not required for readers to get into the weeds of sexual entrapment, the authors might consider getting rid of this sentence. Also, what is the “relevant literature” under the validity and reliability of instrument section?

 (b) Reference no. 20 does not support the claim that the authors stated in page 8. This is because this study was conducted in India, not in Kenya; and it examined the levels of students’ knowledge, not their sexual harassment experiences.

 Please do check all references closely to present them accurately.   

 (c) What is the difference between reference numbers 16 and 17? They appear to be the same except for their hyperlinks. In these references, to clear the ambiguity, the authors might introduce a sub-title, indicating their content such as questionnaire, in-depth guideline, code, or data set, as appropriate.

Is the work clearly and accurately presented and does it cite the current literature?

Partly

If applicable, is the statistical analysis and its interpretation appropriate?

Partly

Are all the source data underlying the results available to ensure full reproducibility?

Yes

Is the study design appropriate and is the work technically sound?

Yes

Are the conclusions drawn adequately supported by the results?

Partly

Are sufficient details of methods and analysis provided to allow replication by others?

Partly

Reviewer Expertise:

Gender-based violence, adolescent sexual and reproductive health, and maternal health using quantitative and qualitative research methods with the lenses of feminist and intersectionality theories.

I confirm that I have read this submission and believe that I have an appropriate level of expertise to confirm that it is of an acceptable scientific standard, however I have significant reservations, as outlined above.

# Peer review: Reviewer response for version 1

**DOI:** 10.21956/gatesopenres.14059.r31792

**Published:** 2022-03-07

**Authors:** Andreas Witt

**Affiliations:** 1 University of Ulm, Ulm, Germany

Generally, the manuscript is well written and informative. The methods seem sound.

Introduction:

 The introduction reads well and includes information about what sexual entrapment is and a rationale for how first year students are vulnerable. What I am missing is information about the magnitude of the problem, probably in comparison to international data and more importantly a clear scope or aim of the present research. Also, while sexual entrapment might be the broader focus of the present manuscript, there are many parallels to the sexual abuse and sexual harassment literature that should be considered in the introduction as well as in the discussion section.  

Methods:

 The authors offer a lot of information on the University, which seems less important for the current study, but do not provide important information such as:

1. You asked about psychological trauma; did you offer some type of support to participants?
2. Sampling procedure: how were the (potential) participants approached? How many were eligible? How many declined? How representative is the sample? How many participated in the quantitative study? Who conducted the interviews? Were the interviews transcribed? Who conducted the thematic analyses of the interviews? Were there independent raters?
3. Instruments used: It would be nice for the reader to have a description of the instrument used in the text. What were the variables and how were the psychometrics? The authors report that the instrument showed “a reliability coefficient of 0.97”, but I doubt that the instrument is only one scale. There are several themes explored with the instrument. Additionally, the knowledge questions about sexual entrapment seem a little suggestive.  
4. You report that Chi-square statistics were done, but there are no results reported in the manuscript, but then referred to in the discussion section.

Results:

1. How was “good”, “fair” and “poor” knowledge operationalized? Such information should be included in the methods section.
2. “40.8% of the respondents’ experienced sexual entrapment before coming to the university followed by 39.5% while in 100 level” is unclear to me.

Discussion:

1. The discussion should include a section about the limitations of the study. While there are many strengths, there are a couple of limitations (e.g. social desirability in answers, etc.).
2. In my opinion the discussion should not only discuss whether the findings are in line with other studies, but also how they can and should be used to for example design prevention strategies or to offer support for victims.

Is the work clearly and accurately presented and does it cite the current literature?

Partly

If applicable, is the statistical analysis and its interpretation appropriate?

Yes

Are all the source data underlying the results available to ensure full reproducibility?

Partly

Is the study design appropriate and is the work technically sound?

Yes

Are the conclusions drawn adequately supported by the results?

Yes

Are sufficient details of methods and analysis provided to allow replication by others?

Partly

Reviewer Expertise:

Trauma, child maltreamtment, interventions, out of home placement

I confirm that I have read this submission and believe that I have an appropriate level of expertise to confirm that it is of an acceptable scientific standard.

# Peer review: Reviewer response for version 1

**DOI:** 10.21956/gatesopenres.14059.r27587

**Published:** 2019-08-22

**Authors:** Caleb Adegbenro

**Affiliations:** 1 Department of Community Health, College of Health Sciences, Obafemi Awolowo University Teaching Hospital, Ile-Ife, Nigeria

The following are my comments:

 1) “Sexually Transmitted Diseases (STDs)” should be changed to "Sexually Transmitted Infections (STIs)" so as to conform with the current trend globally.

 2) Under Study setting:

- Line 8 from the top, the word "called" University of Ibadan in 1962 should be replaced by "changed" to University of Ibadan in 1962.
- Line 4 from the bottom, the whole sentence, "The University has several social facilities that that can aid as a medium to sexually entrap on campus…" should be changed to "The University has several social facilities that can serve as a medium to sexual entrapment on campus…".

3) Under Sampling procedure:

- These sections should be arranged by putting Sample size Determination first.
- Leslie Kish's formula was not referenced both in the body of your write up and in the full referencing.
- The word "respondents," should be written as "Respondents'” i.e. putting an apostrophe at end of "s".

4) Data management, analysis and presentation:

- There was no information on the criteria for classifying "Knowledge" into either, "poor" or "fair" or "good" knowledge. Also, the criteria for classifying "Perception" into either "Negative" or "Positive" perception.

5) Results:

- Under Table 3: The level of "Perception" should either be "Negative" or "Positive" perception and NOT "Good" or "Poor" perception.

General comment: You should be consistent on the use of "Respondents" and "Study Participants". You should stick to only one of the two.

Is the work clearly and accurately presented and does it cite the current literature?

Yes

If applicable, is the statistical analysis and its interpretation appropriate?

Yes

Are all the source data underlying the results available to ensure full reproducibility?

Yes

Is the study design appropriate and is the work technically sound?

Yes

Are the conclusions drawn adequately supported by the results?

Yes

Are sufficient details of methods and analysis provided to allow replication by others?

Yes

Reviewer Expertise:

NA

I confirm that I have read this submission and believe that I have an appropriate level of expertise to confirm that it is of an acceptable scientific standard.
